# Breast elastography: diagnostic performance of computer-aided diagnosis software and interobserver agreement

**DOI:** 10.1590/0100-3984.2019.0035

**Published:** 2020

**Authors:** Eduardo F. C. Fleury, Karem Marcomini

**Affiliations:** 1 Faculdade de Ciências Médicas da Santa Casa de São Paulo, São Paulo, SP, Brazil.; 2 IBCC - Instituto Brasileiro de Controle do Câncer, São Paulo, SP, Brazil.

**Keywords:** Ultrasonography, Elasticity imaging techniques, Breast, Diagnosis, computer-assisted, Observer variation

## Abstract

**Objective:**

To determine the best cutoff value for classifying breast masses by ultrasound elastography, using dedicated software for strain elastography, and to determine the level of interobserver agreement.

**Materials and Methods:**

We enrolled 83 patients with 83 breast masses identified on ultrasound and referred for biopsy. After B-mode ultrasound examination, the lesions were manually segmented by three radiologists with varying degrees of experience in breast imaging, designated reader 1 (R1, with 15 years), reader 2 (R2, with 2 years), and reader 3 (R3, with 8 years). Elastography was performed automatically on the best image with computer-aided diagnosis (CAD) software. Cutoff values of 70%, 75%, 80%, and 90% of hard areas were applied for determining the performance of the CAD software. The best cutoff value for the most experienced radiologists was then compared with the visual assessment. Interobserver agreement for the best cutoff value was determined, as were the interclass correlation coefficient and concordance among the radiologists for the areas segmented.

**Results:**

The best cutoff value of the proportion of hard area within a breast mass, for experienced radiologists, was found to be 75%. At a cutoff value of 75%, the interobserver agreement was excellent between R1 and R2, as well as between R1 and R3, and good between R2 and R3. The interclass concordance coefficient among the three radiologists was 0.950. When assessing the segmented areas by size, we found that the level of agreement was higher among the more experienced radiologists.

**Conclusion:**

The best cutoff value for a quantitative CAD system to classify breast masses was 75%.

## INTRODUCTION

The use of artificial intelligence as an auxiliary tool for classifying breast masses detected by imaging methods is currently under discussion. With the aim of optimizing interobserver agreement and diagnostic accuracy, technologies such as deep learning and machine learning are being tested^(1-4)^.

For strain elastography, the main limitation reported is the lack of standardization of the technique and, most importantly, the lack of defined criteria for the final classification of lesions. In addition, poor interobserver agreement could be a major limitation^(5,6)^.

Some authors have reported that a dedicated strain elastography computer-assisted diagnosis (SE-CAD) system is able to classify masses by means of color stratification^(7,8)^. According to the lexicon in the 5th edition of the Breast Imaging Reporting and Data System (BI-RADS), masses can be classified by elastography as soft, intermediate, or hard, although there is no clear standardization of the classification^(9)^.

As proposed in the BI-RADS lexicon, hard lesions are typically associated with malignancy, whereas soft and intermediate lesions should be considered as benign. The reason for this hard aspect is that malignancy is most often associated with high cellularity and intense peritumoral desmoplasia. Therefore, hard masses are considered positive for malignancy, whereas intermediate and soft masses are considered negative.

Determination of the best cutoff value is extremely important for the classification of masses, given that it will be the standard for software operation and will provide the best diagnostic accuracy. The aim of this study was to determine the cutoff value for a dedicated SE-CAD system that allows the best classification of masses according to the BI-RADS lexicon.

## MATERIALS AND METHODS

We evaluated 83 consecutive breast masses in 83 patients referred for percutaneous breast biopsy between March and May of 2016. On the basis of the histological analysis, 31 of those masses had been categorized as malignant and 52 had been categorized as benign. The study was approved by the local research ethics committee (Protocol no. 012664/2016), and all patients gave written informed consent. Between January and March of 2017, we evaluated newly released software, testing several cutoff values for classifying the masses.

Three radiologists, all with experience in elastography, participated in the study: one with 15 years of experience, designated reader 1 (R1); one with 2 years of experience, designated R2; and one with 8 years of experience, designated R3. All images were acquired by R2, who employed a Toshiba ultrasound system (Aplio 300; Toshiba, Tokyo, Japan), and were analyzed with commercially available software for strain elastography. After B-mode scans had been acquired, elastography was performed as previously described^(10,11)^. In brief, we used the first compression-decompression cycle, during which we can determine lesion stiffness on the basis of the cumulative energy, to obtain the elastography image. The native ultrasound elastography software has image quality criteria that allow the best image to be selected. The images obtained were imported into a picture archiving and communication system. On the basis of the elastography images, the radiologists gave each lesion a score of 1 (soft), 2 (intermediate), or 3 (hard). All patients underwent percutaneous biopsy, and the results were used as the gold standard for comparative purposes.

**Figure 1:**
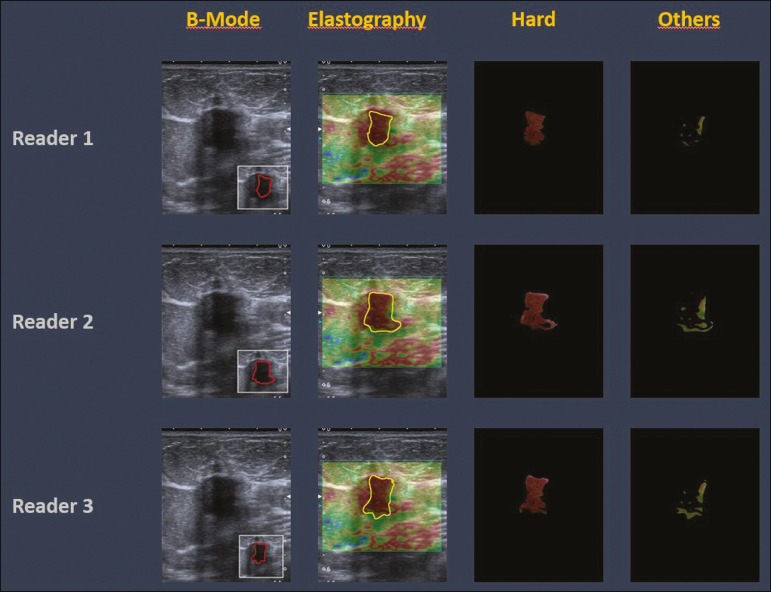
Example of image stratification by reader, showing the area segmented by each radiologist. In B-mode images, the lesion was manually segmented by the readers (small frame). This information was automatically transported to the elastography image. The software extracts and separates the hard and soft components of the lesion. The red outline indicates the area segmented by the radiologist. It is of note that the radiologist with more experience (reader 1) segmented an area smaller than that segmented by the radiologist with less experience (reader 3). The outlined area is automatically transferred to the elastography image without any intervention by the radiologist. The hard area is separated from the others.

The three radiologists, all of whom were blinded to the elastography and histological results, evaluated the images. An SE-CAD system was used for classification of the masses. Each radiologist manually segmented the mass in the B-mode image. The software automatically transports the selected area to the elastography image, where it is stratified by rigidity (Figure 1).

### SE-CAD color stratification

Elastography images were converted from the red-green-blue (RGB) color space to the Commission Internationale de l’Éclairage L*a*b* (CIELab) color space (where L* is the lightness from black to white, a* is the range from green to red, and b* is the range from blue to yellow), in order to extract the corresponding hard areas, as described in previous studies^(7,8)^. To standardize the stratification, we adopted red to indicate hard lesions and blue to indicate soft lesions, a strategy we believe to be the most intuitive. After the images had been converted from the RGB to the CIELab color space, the Otsu method was applied in the a* channel to define and quantify hard areas^(8)^. Masses were classified according to the proportion of hard area within the mass, adopting *Z* as the cutoff value, where the *Z*s tested were 70%, 75%, 80%, and 90%^(12)^. In the final analyses, the masses were classified as follows: soft (< 50% of hard area); intermediate (50-*Z*% of hard area); or hard (> *Z*% of hard area).

From the area segmented by the radiologist, the SE-CAD system classified the masses automatically. One month after the radiologists performed the SE-CAD classification, the lesions were classified according to the visual pattern, following the classification guidelines proposed in previous studies^(11,13)^. The visual classification also consists of three categories based on the proportion of hard area within the mass: soft (< 50%); intermediate (50-90%); and hard (> 90%). The three radiologists were also blinded to the SE-CAD results, and the final visual classification was determined by consensus.

Diagnostic accuracy, sensitivity, and specificity, together with the area under the receiving operating characteristic curves, were calculated for each radiologist. For the SE-CAD and visual classifications, interobserver agreement was assessed by calculating Cohen’s kappa statistic, the intraclass correlation coefficient (ICC), and the concordance correlation coefficient (CCC). For Cohen’s kappa statistic, the CCC, and the ICC, interobserver agreement was classified as slight (0.0-0.2), fair (0.2-0.4), moderate (0.4-0.6), substantial (0.6-0.8), or excellent (0.8-1.0). Values of *p* < 0.05 were considered statistically significant.

The radiologist-segmented areas were also compared. Areas were considered equal when the variation between two radiologists was less than 20%. When areas were considered unequal, they were categorized as smaller or larger. When the product of dividing one area by the other was < 0.8, the area was categorized as smaller, whereas it was categorized as larger when the product was > 1.2. For statistical analysis, we used MedCalc Statistical Software, version 19.1.3 (MedCalc Software, Ostend, Belgium).

## RESULTS

At the beginning of the study, we chose to compare cutoff (*Z*) values of 70%, 80%, and 90%. The initial results indicated that a *Z* of 70% had the best diagnostic accuracy (Table 1). Because of the wide range between *Z* values of 70% and 80%, we decided to perform a test with an intermediate *Z* of 75%, which improved diagnostic accuracy for the more experienced radiologists (R1 and R3). The best diagnostic accuracy was achieved by R1, followed by R3 and R2, although the difference was not statistically significant at *p* < 0.005. There was also no statistically significant difference between the SE-CAD and visual classifications for any of the three radiologists. The computation required in order to obtain the study data is of low complexity, and the results are produced almost instantaneously (in less than 1 s).

**Table 1:** Area under the ROC curve, sensitivity, and specificity for R1, R2, and R3 at cutoff values of 70%, 75%, 80%, and 90%. The same parameters are shown for the visual classification (at a cutoff value of 75%).

	Cutoff value														
	70%				75%				80%				90%		
Reader	AUC	Sensitivity	Specificity		AUC	Sensitivity	Specificity		AUC	Sensitivity	Specificity		AUC	Sensitivity	Specificity
R1	0.841	80.6	80.8		**0.853**	**71.0**	**88.5**		0.802	96.8	48.1		0.790	96.8	48.1
R2	**0.833**	**80.6**	**82.3**		0.806	67.7	84.6		0.815	54.9	94.2		0.707	93.5	44.2
R3	0.802	71.0	80.8		**0.814**	**58.0**	**90.4**		0.789	93.5	48.1		0.723	93.5	48.1
Visual					**0.829**	**90.3**	**63.5**								

AUC, area under the curve. Obs.: Values in bold indicate statistical significance.

When a cutoff value of *Z* = 75% was applied, the level of interobserver agreement for the SE-CAD classification, as determined by calculating Cohen’s kappa statistic, was excellent between R1 and R2, as well as between R1 and R3, and was strong between R2 and R3. When the same cutoff value was applied to the visual classification, the level of interobserver agreement was strong for all of the readers (Table 2). The CCC and ICC results demonstrated excellent agreement between all of the readers (Table 3).

**Table 2:** Cohen's kappa statistic for interobserver agreement among R1, R2, and R3, as well as a comparison with a visual classification.

		R1					R2					R3			
Reader		70%	75%	80%	90%		70%	75%	80%	90%		70%	75%	80%	90%
R2	70%	0.830													
	75%		0.800												
	80%			0.794											
	90%				0.685										
R3	70%	0.831					0.708								
	75%		0.808					0.665							
	80%			0.798					0.667						
	90%				0.733					0.603					
Visual		0.662	0.679	0.675	0.601		0.705	0.698	0.713	0.487		0.703	0.754	0.736	0.604

**Table 3:** CCC and ICC for all of the readers.

	CCC		
Reader	R1	R2	ICC
R2	0.8720		
R3	0.8861	0.8278	
All			Average 0.9503
			Single 0.88644

When analyzing the radiologist-segmented areas, we found that agreement was best between R1 and R2, at 83.1%, compared with 78.3% between R1 and R3 and 72.3% between R2 and R3 (Table 4).

**Table 4:** Concordance between the radiologists (readers) in terms of the size of the areas segmented.

Areas delimited (x/y)	Equal areas (x = y)	Smaller areas (x < y)	Larger areas (x > y)
R1/R2	69 (83.1%)	5 (6.0%)	9 (10.8%)
R1/R3	65 (78.3%)	7 (8.4%)	11 (13.2%)
R2/R3	60 (72.3%)	9 (10.8%)	14 (16.9%)

**Figure 2:**
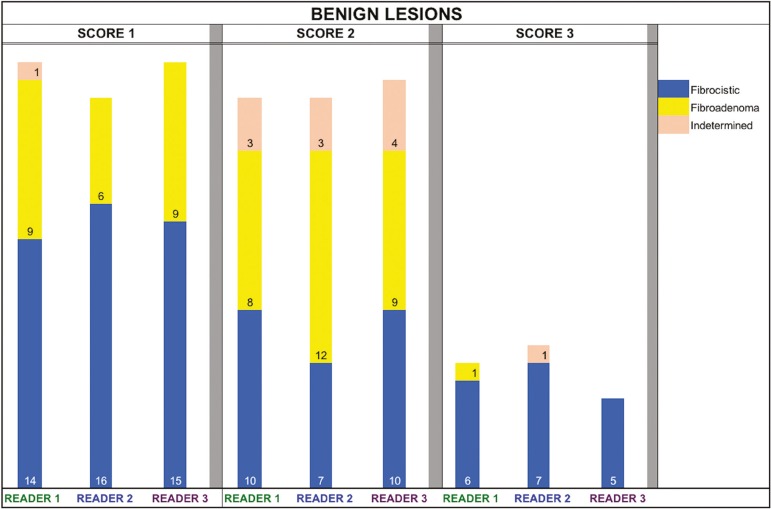
Distribution of benign lesions for readers 1, 2, and 3, according to the elastography score: 1 indicates a soft lesion; 2 indicates an intermediate lesion; and 3 indicates a hard lesion. The absolute numbers for each of the most common lesions, as determined by histology, are shown within the bars, where blue indicates a fibrocystic change, yellow indicates a fibroadenoma, and pink indicates an indeterminate lesion, such as a papillary lesion.

**Figure 3:**
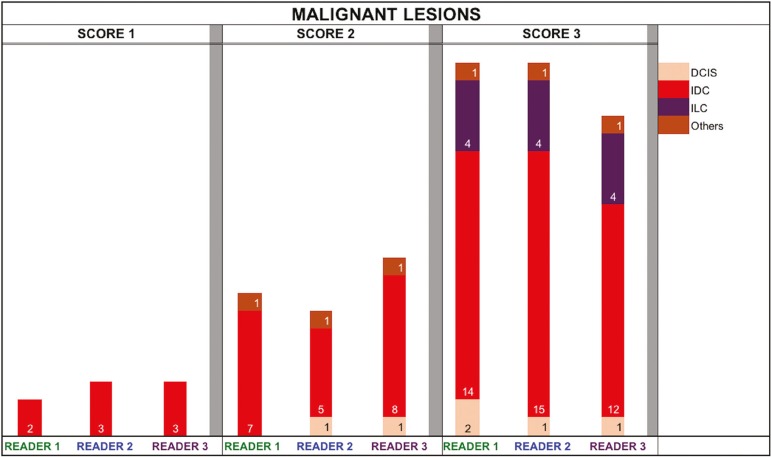
Distribution of malignant lesions for readers 1, 2, and 3 according to the elastography score: 1 indicates a soft lesion; 2 indicates an intermediate lesion; and 3 indicates a hard lesion. The absolute numbers for each of the most common lesions, as determined by histology, are shown within the bars, where pink indicates ductal carcinoma in situ, red indicates invasive ductal carcinoma, purple indicates invasive lobular carcinoma, and brown indicates other malignant neoplasms, such as mucinous and papillary carcinomas.

Figures 2 and 3 present the distribution, by radiologist score, of the lesions histologically classified as benign and malignant, respectively. For comparative purposes, benign lesions were divided into fibroadenoma, fibrocystic changes, and indeterminate lesions, such as papillary lesions and atypical ductal hyperplasia (Figure 2). Malignant lesions were divided into invasive ductal carcinoma, invasive lobular carcinoma, ductal carcinoma in situ, and other malignant neoplasms, such as papillary carcinomas and mucinous carcinoma (Figure 3).

## DISCUSSION

In clinical practice, the main limitations of breast ultrasound are poor interobserver agreement and limited reproducibility of the results. With the introduction of digital technology and the recent revolution in the areas of technology and computer science, the application of CAD systems came to be studied as an aid in the diagnosis of breast lesions^(14,15)^.

Breast ultrasound is considered to have low specificity, which limits its implementation in breast cancer screening programs. The introduction of breast elastography findings into the BI-RADS lexicon increased the specificity of the examination, resulting in better diagnostic performance. However, poor interobserver agreement and limited reproducibility continue to restrict the utility of the technique^(16)^.

Through the use of CAD classification systems for breast masses, it is possible to standardize the classification criteria and improve interobserver agreement. Recent studies have shown that CAD systems can bring the performance of less experienced readers closer to that of those with more experience. That is especially due to the fact that such systems do not use subjective criteria, as are typically employed in the visual classification of breast masses^(13,16,17)^.

Currently, strain elastography is the most widely available and affordable elastography method in clinical practice. However, criticism regarding its reproducibility and lack of standardization in the classification of breast masses has discouraged its use^(18)^. A CAD system could overcome that limitation. Optimized results of a CAD system are directly related to its calibration; the better calibrated it is, the better its results will be. For the evaluation of breast masses, strain elastography and shear wave elastography have similar sensitivity and specificity. However, shear wave elastography is limited in its ability to evaluate superficial lesions and is less widely available because it is more expensive. For superficial organs, strain elastography is preferred, whereas shear wave elastography is more appropriate for deep organs such as the liver.

Some studies have used CAD systems to classify breast masses by elastography, although without employing the BI-RADS lexicon classification and without comparing the best cutoff values^(7,18)^. In the study conducted by Zhang et al.^(7)^, a cutoff value of 80% hard areas was used but the authors adopted a 5-point classification system^(7,19)^. In the present study, cutoff values of 70%, 80%, and 90% hard areas were initially tested in the areas segmented by the radiologists. In the first analysis, the best results were obtained with a cutoff value of 70%. An additional test was then performed with an intermediate cutoff value of 75%, which was found to provide the best diagnostic accuracy.

In the present study, manual segmentation of breast masses was used because we believe that classification of breast masses is under the purview of the physician^(13)^. In using the software, radiologists were instructed to segment the area of the mass that was most hypoechoic, delineating the area immediately inside the margins displayed in B mode. The objective was to minimize contamination of the sample by normal breast tissue. We found good agreement among the readers in terms of the areas segmented. Training radiologists to delineate only areas of lower echogenicity, respecting the margins, could contribute to improving the diagnostic accuracy of CAD systems. It is also noteworthy that the lesions were delineated after the ultrasound examination, during the postprocessing period, when the radiologist writes the final report, and the scan time was therefore not increased. The time spent delineating the lesions was similar (< 10 s) for all three radiologists.

For the most experienced radiologist (R1), diagnostic accuracy was better when the SE-CAD system was used than when the consensus visual classification was used. For R2 and R3, diagnostic accuracy was lower when the consensus visual classification was used, although the differences were not statistically significant. This shows uniformity in the final classification by the readers. The high levels of agreement among the readers demonstrate the applicability of the method to improve reproducibility and standardize classifications, especially for less experienced readers. The use of CAD software to classify lesions may improve diagnostic accuracy for less experienced radiologists and make the results more homogeneous.

When analyzing the distribution of the lesions according to the classification given by each reader, we observed that the second most experienced reader (R3) classified fewer malignant lesions as having a score of 3 than did the other readers, although the difference was not statistically significant. That is due to the complex appearance of malignant lesions on ultrasound images, on which it is often difficult to distinguish between healthy and pathological tissue. As can be seen in Figure 3, some malignant lesions were classified as soft (with a score of 1). All of the carcinomas classified as soft were high-grade lesions containing areas of necrosis. It should be borne in mind that elastography does not differentiate benign lesions from malignant lesions, rather differentiating between soft and hard tissue. High-grade carcinomas with necrosis are expected to be classified as soft lesions, whereas low-grade lesions accompanied by desmoplasia are expected to be classified as hard. However, all of the false-negative elastography results were for tumors with a morphology that had raised the suspicion of malignancy on B-mode ultrasound. In such cases, the morphology of the lesion should be considered in conjunction with the elastography findings in order to make the final classification in accordance with the BI-RADS lexicon.

Elastography is a tool that is complementary to ultrasound in the evaluation of breast masses. It should not be used, in isolation, to differentiate between benign and malignant lesions. However, combining elastography results with those of ultrasound can improve the performance of ultrasound in the diagnosis of breast masses.

Our study has some limitations. First, all of the images were acquired by the same radiologist. That is due to the study objective, which was not to standardize the technique (which has been widely studied) but to standardize the classification system. The least experienced radiologist was elected, in order to demonstrate the ease of obtaining quality images by strain elastography. Another limitation is the relatively small sample size. However, we opted for prospective, consecutive image acquisition, which accurately reproduces the daily clinical routine at our institution. An additional limitation is that the consensus final visual classification adopted as the gold standard might have introduced a bias of the more experienced over the less experienced.

Our results demonstrate that the use of a cutoff value of 75% for the SE-CAD system of classifying breast masses provides high diagnostic accuracy and interobserver agreement among radiologists.
